# Selection from a Clinical Lecture

**Published:** 1891-02

**Authors:** Wm. Goodell

**Affiliations:** Philadelphia


### Selection from a Clinical Lecture,

BY WM. GOODELL, M.D., OF PHILADELPHIA.

We now ask ourselves, What are dermoid cysts? This is a question in regard to which pathologists have not yet become satisfied. We generally find in them hairs—most commonly of a light color—and teeth. We sometimes find in them bone fragments, but the most common constituents are hairs, teeth, and cheesy material such as comes from sebaceous glands, viz: skin products, and it is for this reason that these curious growths are called *dermoid* cysts—in other words, skin-like tumors.

For a long time they were explained by what I call the "blasted theory"—partly because I can not understand it, and partly because it contends that the epiblastic portions of an ovum, which form the skin, hair, and teeth, become misplaced by dipping down into the hypoblast and becoming mixed up with other embryonic cells. Dermoid cysts are sometimes found in the testicles, but more commonly in the ovary, so that the theory is now gaining ground that they are, as a rule, developed from some addled human eggs, and it is no longer considered necessary, as it once was, that an ovum should have been fructified by the spermatozoa in order for it to become the basis of a dermoid cyst.

Another theory once in vogue was that of parthenogenesis, or virgin birth; that is to say, imperfect effort at transmitted fertility—a property peculiar to many insects—the aphidæ, for instance—one copulation being sufficient to keep up fructification for several generations of decendants. Let us here take the opportunity, while I am on this subject, to correct the statement I made in the beginning of my lecture that this trouble in the case before us was probably of specific origin, as this tumor abundantly explains all the symptoms without an appeal to syphilis.

The Dental Protective Association is increasing in numbers constantly. Every honest dentist should be a member, as it will insure immunity from senseless litigation.

